# Molecular Dynamics Simulations of Water/Mucus Partition Coefficients for Feeding Stimulants in Fish and the Implications for Olfaction

**DOI:** 10.1371/journal.pone.0072271

**Published:** 2013-09-02

**Authors:** Alex D. Rygg, Adri C. T. van Duin, Brent A. Craven

**Affiliations:** 1 Department of Mechanical and Nuclear Engineering, The Pennsylvania State University, University Park, Pennsylvania, United States of America; 2 Applied Research Laboratory, The Pennsylvania State University, University Park, Pennsylvania, United States of America; 3 Department of Bioengineering, The Pennsylvania State University, University Park, Pennsylvania, United States of America; Wake Forest University, United States of America

## Abstract

The odorant partition coefficient is a physicochemical property that has been shown to dramatically influence odorant deposition patterns in the mammalian nose, leading to a chromatographic separation of odorants along the sensory epithelium. It is unknown whether a similar phenomenon occurs in fish. Here we utilize molecular dynamics simulations, based on a simplified molecular model of olfactory mucus, to calculate water/mucus partition coefficients for amino acid odorants (alanine, glycine, cysteine, and valine) that are known to elicit feeding behavior in fish. Both fresh water and salt water environments are considered. In fresh water, all four amino acids prefer the olfactory mucus phase to water, and the partition coefficient is shown to correlate with amino acid hydrophobicity. In salt water, a reversal in odorant partitioning is found, where each of the feeding stimulants (except glycine) prefer the water phase to olfactory mucus. This is due to the interactions between the salt ions and the odorant molecules (in the water phase), and between the salt and simplified mucin (in the olfactory mucus phase). Thus, slightly different odorant deposition patterns may occur in the fish olfactory organ in fresh and salt water environments. However, in both underwater environments we found that the variation of the water/mucus odorant partition coefficient is approximately one order of magnitude, in stark contrast to air/mucus odorant partition coefficients that can span up to six orders of magnitude. We therefore anticipate relatively similar deposition patterns for most amino acid odorants in the fish olfactory chamber. Thus, in contrast to terrestrial species, living in an underwater environment may preclude appreciable chromatographic odorant separation that may be used for spatial coding of odor identity across the olfactory epithelium. This is consistent with the reported lack of spatial organization of olfactory receptor neurons in the fish olfactory epithelium.

## Introduction

The partition coefficient is a physicochemical property that is important in a wide variety of applications. Physically, the partition coefficient represents the relative affinity of a chemical for two different solvents. Mathematically, it is defined as the concentration ratio of a chemical in two phases at equilibrium, as described by the Nernst distribution (or partition) law [1], [2]

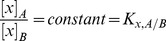
(1)where 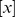 is the concentration of a solute 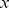 in phase 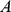 or 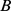, and 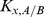 is the 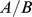 partition coefficient of 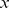. While this parameter is important in a number of fields (e.g. environmental pollution [3], drug delivery [4], [5], and geochemistry [6]), we are interested in determining the partitioning behavior of feeding stimulants in the olfactory mucus of fish and the potential implications for underwater olfaction.

In mammalian olfaction, the air/mucus partition coefficient of odorants has been shown to dramatically influence odorant deposition patterns in the olfactory region of the nose, wherein highly-soluble odorants are deposited anteriorly in the sensory region and less-soluble odorants are deposited more uniformly [7]. Such odorant-dependent deposition patterns result in a “chromatographic” separation of odorants along the sensory epithelium [8]–[13], which has been shown to broadly correspond with the spatial organization of olfactory receptor neurons (ORNs) [14]–[17] in the sensory region of the nasal cavity [7], [18]. That is, as odorant-laden air flows through the olfactory region of the nose (e.g., see [19]), an odorant is deposited in a region containing ORNs that are sensitive to that specific class of odorant. Such “imposed” odorant deposition patterns are used by the “inherent” distribution of ORNs for improved olfactory discrimination [11], [13].

It is unknown whether a similar phenomenon occurs in fish as water-borne odors circulate through the olfactory chamber. Recent computational fluid dynamics (CFD) simulations of water flow in an anatomically-accurate reconstruction of the head and olfactory chamber of the hammerhead shark [20] were used to elucidate the external and internal hydrodynamics of olfaction during swimming. Simulations of odorant transport are planned, which require odorant partition coefficients. However, though values have been published for many different solvents (e.g., [4], [21]–[24]), water/mucus odorant partition coefficients do not exist.

The objective of this study is to use molecular dynamics simulation techniques [6], [25]–[28] to calculate the water/mucus partition coefficients for underwater odorants, namely chemicals known to elicit feeding behavior in fish (i.e., feeding stimulants). We begin by reviewing the morphology of the olfactory organ of a representative cartilaginous fish, the hammerhead shark, which contains numerous lamellae that are lined with sensory epithelium, covered by a thin, viscous mucus layer. A summary of the evidence for the existence of the olfactory mucus layer in fish is provided, followed by the formulation of a simplified model of olfactory mucus. This model is subsequently used in molecular dynamics simulations to calculate the water/mucus odorant partition coefficients in fresh and salt water. Finally, the implications of the results regarding fish olfaction are discussed.

## Methods

### Physical Model

#### Ultrastructure of the Olfactory Epithelium

**Figure 1:**
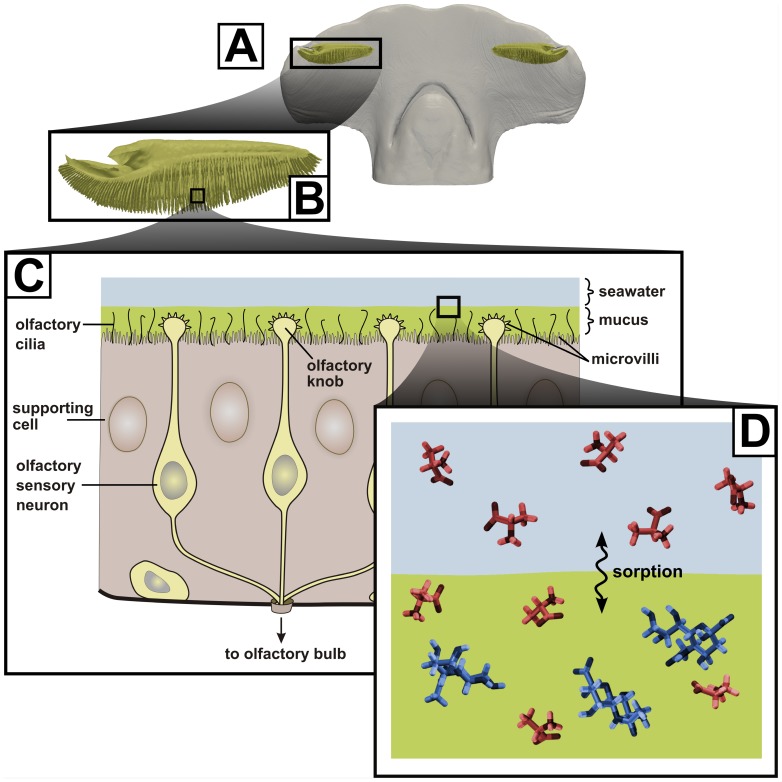
Multi-scale view of olfaction in the hammerhead shark. The hammerhead shark (A) possesses two complex olfactory organs that contain numerous lamellae, as shown in B. The ultrastructure of the sensory epithelium that covers the lamellae is illustrated in C. At the nanoscale (D), sorption of odorant molecules (shaded in red) takes place at the seawater/mucus interface. In the mucus phase, these odorant molecules likely interact with mucin sugar molecules (shaded in blue). Note: the panels are for illustrative purposes and are not drawn to scale.

The olfactory chamber of the hammerhead shark (Figure 1 A and B) contains two rows of olfactory lamellae that provide an increased surface area for chemical sensing [20], [29]. Functionally, as the shark swims, water flows through the olfactory chamber, as described by Rygg et al. [20], delivering odorant to the sensory epithelium that lines each lamella (Figure 1 C). Previous studies [30]–[33] have characterized the sensory epithelium in elasmobranch fishes (sharks, rays, and skates). At the ultrastructural level, supporting cells separate the olfactory sensory neurons, which project a dendritic process to the epithelial surface that terminates in an expanded olfactory knob (Figure 1 C). Microvilli are present on both the supporting cells and the olfactory knobs of the sensory neurons. Non-sensory motile cilia originate from the supporting cells, and in elasmobranchs (unlike in teleosts) the olfactory sensory neurons are not ciliated [30]–[35].

#### Olfactory Mucus Layer

According to Getchell and Getchell [36], “the surface of the olfactory sensory epithelium of aquatic and terrestrial vertebrates is covered by a layer of mucus in which odorant molecules dissolve prior to reaching the site of olfactory stimulation, the cilia of the olfactory receptor neurons” (or the microvilli in elasmobranchs). Generally, the mucus layer serves a number of functions, including the protection of the olfactory epithelium from exposure to noxious chemicals, metabolism and removal of odorants, and to provide the biochemical microenvironment for olfactory transduction [37], [38]. In fish, olfactory mucus is produced by goblet cells [30], [31], [34], [36], [39], and an olfactory mucus layer has been observed on the olfactory lamellae of the sea trout [40], zebrafish [41], [42], freshwater minor carp [43], and the clearnose skate [34] (an elasmobranch).

In the respiratory system of terrestrial vertebrates, nasal mucus is comprised of a viscous superficial ‘gel’ layer and an underlying aqueous ‘sol’ (or periciliary) layer [44]–[46]. In contrast, the mucus that covers the olfactory epithelium in air-breathing animals consists of a thin, superficial watery layer and a thicker, underlying filamentous viscous layer [36]–[38], [47]–[49]. The superficial watery layer is thought to be produced by Bowman's glands [36], [37], [50]. However, Bowman's glands are not present in the olfactory mucosa of fish [30], [34], [36]. Further, water flowing through the nasal chamber would wash off such a superficial watery layer produced by other means. Therefore, at the macroscale, the olfactory mucus layer in fish may be well approximated by a filamentous viscous mucoid layer that is not washed off by low-speed water flowing through the olfactory chamber (e.g., see [20]).

#### Molecular Description of Olfactory Mucus

Olfactory mucus gets most of its properties from its high water content (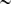
80–95% [45], [51]) and large macromolecules known as glycoproteins (or mucins), which give it the viscous consistency of a “gel” [52]. On a molecular level, mucins are typically 0.1 to 5 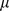
m in length [51] and contain a polypeptide core that is largely glycosylated [45], [51]. Due to this heavy glycosylation, mucins are composed of approximately 75% carbohydrates and 25% amino acids [51]. Apomucin (i.e., the unglycosylated peptide core) is insoluble in water, and therefore it is the carbohydrate chains that confer hydrophilic properties to the protein and allow for hydration of the mucus [51], [53]. Common sugars found in the carbohydrate chains are fructose, galactose, N-acetylglucosamine, N-acetylgalactosamine, and N-acetylneuraminic acid (sialic acid) [45]. It is common for O-linked glycans (which are abundant in mucins) to be capped by sialic acid [54]. Sialic acid, being a negatively charged residue, may contribute to water uptake and retention. Although many of these properties have primarily been investigated in humans and other mammals, mucins from different organs and species have a similar general structure [51], and glycoproteins in fish mucus appear to be similar to those in mammals [52].

### Molecular Models of Olfactory Mucus and Water

Given the foregoing molecular description, we developed a simplified molecular model of olfactory mucus for use in molecular dynamics simulations. Due to the complex composition of olfactory mucus, it is presently impractical to model all of the molecules that comprise the mucus phase. In addition, calculating solvation energies using molecular dynamics works best if the solvent is comprised of relatively small compounds [26]. Therefore, instead of trying to model the complete mucus phase, we aimed to model the most relevant interactions that occur between the mucus and odorant molecules.

To develop a realistic simplified molecular model of the mucus phase, we first assume that olfactory mucus in fish is similar in composition to the viscous mucus layer covering the mammalian olfactory epithelium (see Olfactory Mucus Layer). To further simplify the model, we approximate the mucus phase as being comprised of water and mucins, as water is the primary component of olfactory mucus and mucins are the most prominent macromolecule (see Molecular Description of Olfactory Mucus). Such a simplified water-mucin model retains the primary components of olfactory mucus that confer its physical properties, including a gel-like consistency necessary to prevent the mucus layer from washing off of the olfactory lamellae as water flows through the nasal chamber (e.g., see [20]).

Because the carbohydrate chains are responsible for water retention in mucus [45], [51], [53] and comprise 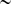
75% of the mucins [51], odorant molecules have a high probability of interacting with the carbohydrates when they come into contact with the mucus phase. Specifically, N-acetylneuraminic acid (sialic acid) is a common terminal monosaccharide on the carbohydrate chains [54], and is therefore most likely to interact with odorant molecules, which are of the same order of size as N-acetylneuraminic acid molecules. Accordingly, based on the above approximations, the olfactory mucus phase was modeled as a solution of 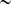
90% water and 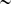
10% N-acetylneuraminic acid.

Finally, to compare differences between underwater environments, we constructed molecular models for both fresh water and salt water. The fresh water phase was straightforward, and consisted of a bath of 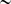 450–550 water molecules. Modeling the salt water phase consisted of solvating each odorant in a bath of water molecules, then adding salt ions up to a salinity of 3.5%, a typical salinity for seawater [55]. Because the water and mucus phases have such similar compositions, we assumed an even distribution of salt between them. That is, in a salt water environment the olfactory mucus phase consisted of water, N-acetylneuraminic acid, and a salt concentration of 3.5%.

### Odorant Chemicals

Unlike olfaction in air, where volatility strongly determines whether a chemical will be detected, underwater olfaction is facilitated by the presence of highly-soluble chemicals with low molecular weight. Amino acids, which are abundant in both tissue and blood [56], [57], have a low molecular weight, are highly water soluble, and are widely regarded as potent feeding stimulants for many different fish [57]–[64]. Thus, four amino acid odorants were selected for comparison in the present study, based on their reported prevalence as feeding stimulants.

According to Carr et al. [57], who analyzed the chemical composition of tissues in a diversity of marine organisms, alanine and glycine are major tissue components and the two most frequently reported feeding stimulants in a variety of fish. In addition, previous studies [65], [66] that utilized the electro-olfactogram (EOG) technique to determine olfactory sensitivity in elasmobranchs showed that hammerhead sharks (*Sphyrna tiburo* and *Sphyrna lewini*) are sensitive to a wide range of amino acids. While interspecific differences were evident, alanine was treated as the “standard” in both studies and was found to elicit a strong olfactory response with a low detection threshold, while glycine elicited a weak-to-moderate EOG response. In the present study, we aim to model amino acids that span a wide range of olfactory detection thresholds. Thus, in addition to alanine and glycine, based on the EOG results reported in [65], [66], cysteine and valine were chosen as amino acids that elicit a moderate-to-strong and weak olfactory response, respectively.

### Molecular Dynamics

**Figure 2:**
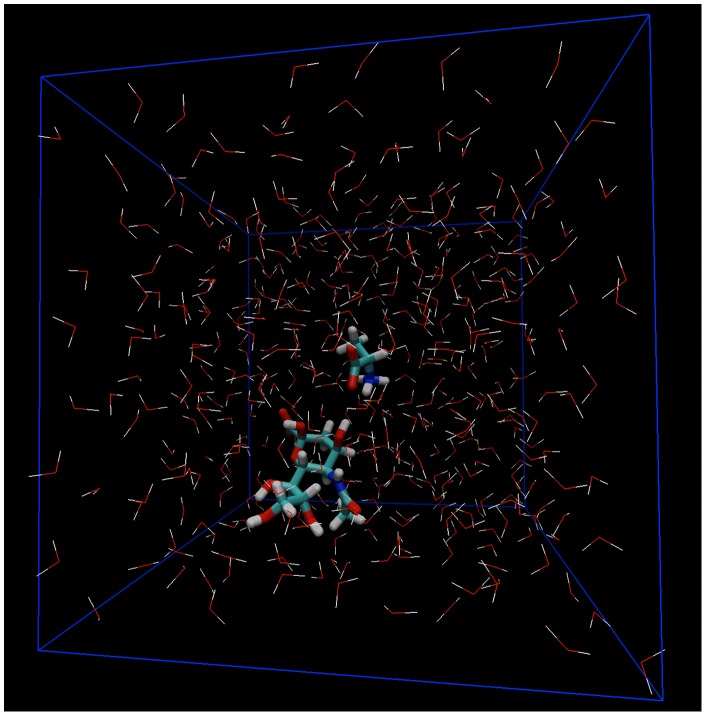
Molecular dynamics model of alanine in mucus. The computational box contains an alanine molecule (the amino acid odorant) immersed in a simplified olfactory mucus phase, consisting of N-acetylneuraminic acid in a bath of water.

Mathematically, the water/mucus partition coefficient of a given molecule, 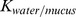, is related to the difference between its solvation energies in each phase, as given by [25], [27]:
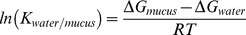
(2)where 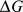 is the solvation energy of the odorant molecule in each phase, 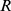 is the ideal gas constant, and 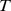 is a reference temperature. Molecular dynamics simulations were used to calculate the solvation energies of each amino acid in the water and mucus phases. Figure 2 shows a representative visualization of the computational box used in the molecular dynamics simulations, which contains an alanine molecule (the amino acid odorant) and N-acetylneuraminic acid surrounded by water (the simplified olfactory mucus phase). Similar molecular dynamics models were constructed for glycine, cysteine, and valine in both fresh water and salt water.

Simulations were performed using the open-source software package GROMACS, version 4.5.4 [67]. The Optimized Potentials for Liquid Simulations All-Atom (OPLS-AA) force field [68]–[71] was used for all molecules, along with the simple point charge water model with included self-energy correction (SPC/E) [72]. A cutoff radius of 1.0 nm was used for the short-range Coulomb interactions, while long-range electrostatics were calculated using the Particle-Mesh Ewald algorithm [73]. Periodic boundary conditions were applied to the computational box in all directions.

Solvation energy can be described as the change in Gibbs free energy when a molecule is transferred from a vacuum to a solvent. In general, the change in Gibbs free energy of a compound between two states, A and B, is given by the following equation [74]:
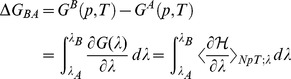
(3)where 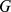 is the Gibbs free energy, 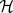 is the Hamiltonian, and 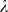 is a coupling parameter that represents the state of the system. Here, 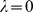 corresponds to a system where the amino acid is completely decoupled from the solvent, while 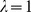 indicates that the amino acid is fully coupled to the rest of the system. Therefore, to calculate the solvation energy of a particular amino acid, Eq. (3) was integrated from 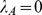 to 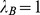. Thermodynamic integration was performed using the Bennett acceptance ratio (BAR) method [75].

Energy minimization of the system was performed using a steepest descent algorithm to a maximum force of 100 kJ mol^−1^ nm^−1^. A second minimization using a low-memory Broyden-Fletcher-Goldfarb-Shanno (BFGS) approach [76] was then performed. The system subsequently underwent 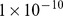 s of constant volume/temperature (NVT) equilibration at a temperature of 300 K, followed by 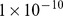 s of constant pressure/temperature (NPT) equilibration at a pressure and temperature of 1 bar and 300 K, respectively. Production molecular dynamics simulations were then run at a constant pressure (1 bar) and temperature (300 K). A Parrinello-Rahman barostat [77], [78] was used, with a pressure coupling time constant of 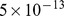 s. A leap-frog stochastic dynamics integrator was utilized in the production molecular dynamics calculations, and temperature coupling was implicitly handled by the integrator. A time constant of 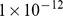 s was used for temperature coupling. Using a time step size of 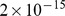 s, simulations were run for 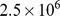 steps, or 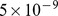 s of simulated time.

### Numerical Accuracy

The OPLS force field was chosen because it has been well validated for the types of molecules and phases that are being modeled in the present study. This force field has been validated with both experimental data and *ab initio* calculations [68]–[70]. Additionally, previous studies [25], [28], [79] have used OPLS to calculate solvation energies and phase behavior for systems involving water/chloroform and octanol/water. The solutes in these studies consisted of n-alkane, polar, nonpolar, alcohol, aromatic, and polychlorinated biphenyl molecules. Originally, OPLS was developed for the simulation of proteins in aqueous solutions [68], and has since been extended to include additional parameters for carbohydrates [70]. Consequently, the OPLS force field was selected in the present work for calculating the solvation energy of amino acids in aqueous solutions containing mucin sugars.

To verify the use of the OPLS-AA force field in our simulations, we calculated the Helmholtz solvation energy of alanine, glycine, cysteine, and valine in pure water using molecular dynamics and compared the values with those reported by Dixit et al. [80], who used the Finite Difference Poisson Boltzmann (FDPB) method (see Table 1). In general, the solvation energies were found to be comparable, with some discrepancy in the absolute values (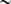 5–10%) that is attributable to the fact that the FDPB method is expected to be less accurate than the molecular dynamics method since the latter considers in more detail the packing of the molecules. Most importantly, however, similar trends in solvation energy were found among the chemicals with both methods (e.g., that cysteine and valine are more hydrophobic than alanine and glycine; see Table 1). Thus, in the absence of experimental data, such a strong comparison between two fundamentally different numerical methods verifies the use of the OPLS-AA force field in the present molecular dynamics simulations of amino acid solvation in aqueous solutions.

**Table 1:** OPLS-AA force field verification.

	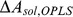	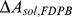 [80]	% Difference
Alanine	−295.44	−323.84	8.77%
Cysteine	−281.50	−311.37	9.59%
Glycine	−299.21	−347.06	13.79%
Valine	−288.63	−298.32	3.25%

Comparison of the Helmholtz solvation energies calculated using the OPLS-AA force field and the Finite Difference Poisson Boltzmann method ([80]).

In terms of accuracy with respect to the physical system, the present molecular dynamics results should not be considered exact due to the approximations made in developing a simplified molecular model of the olfactory mucus phase. Even so, because the simplified molecular model represents the most significant interactions occurring between the amino acid odorant molecules and the mucus phase, the results are expected to be representative of the physical system. Most important to the present study, we expect the relative differences between the calculated water/mucus odorant partition coefficients to be reliable.

## Results

Given the computed solvation energies from the molecular dynamics simulations, partition coefficients were calculated for the four amino acid odorants in both fresh water (Table 2) and salt water (Table 3). In each case, estimates of the numerical error were computed by splitting the simulation data into blocks and calculating the solvation energy for each block. Assuming the data blocks were independent, uncertainty estimates were calculated from the average variance over the blocks [81].

**Table 2:** Molecular dynamics results in fresh water.

	Alanine	Cysteine	Glycine	Valine
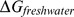 , kJ/mol	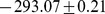	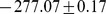	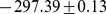	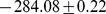
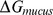 , kJ/mol	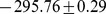	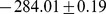	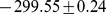	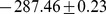
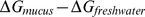 , kJ/mol	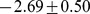	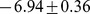	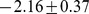	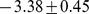
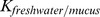	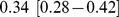	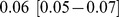	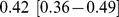	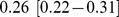

Solvation energies for each amino acid odorant in fresh water and mucus, which were used to calculate the water/mucus odorant partition coefficients.

**Table 3:** Molecular dynamics results in salt water.

	Alanine	Cysteine	Glycine	Valine
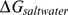 , kJ/mol	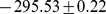	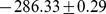	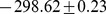	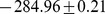
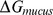 , kJ/mol	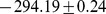	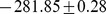	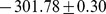	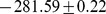
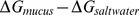 , kJ/mol	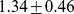	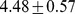	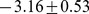	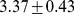
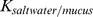	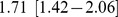	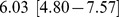	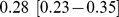	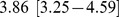

Solvation energies for each amino acid odorant in salt water and mucus, which were used to calculate the salt water/mucus odorant partition coefficients.

The results indicate that the span of the calculated partition coefficients in each underwater environment is approximately one order of magnitude. In the fresh water case (Table 2), all of the partition coefficients are slightly less than one, meaning that all of the amino acids prefer the mucus phase. Moreover, we found that the values of the partition coefficients are correlated with the hydrophobicity of the amino acids. Glycine, which is the most hydrophilic odorant (i.e., it had the lowest value of 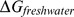), had the largest value of 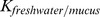. Conversely, cysteine is the most hydrophobic of the four amino acids and also has the smallest partition coefficient. The same trend was similarly observed for alanine and valine. Thus, in fresh water, the more hydrophobic an amino acid, the more readily it is absorbed by the olfactory mucus phase.

In salt water (Table 3), we observed a reversal of the odorant partitioning behavior, where the most hydrophobic amino acids have the largest values of 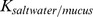. Specifically, we found that the amino acids tend to prefer salt water to fresh water, as indicated by the lower values of 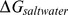 compared to 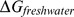. This may be explained by the fact that amino acids in their zwitterionic form likely interact with the salt ions in the water phase due to their internal charges. Additionally, the results indicate that adding salt to the mucus decreases the affinity of the amino acids for this phase – the exception is glycine, which is a very versatile molecule due to its short side chain.

**Figure 3:**
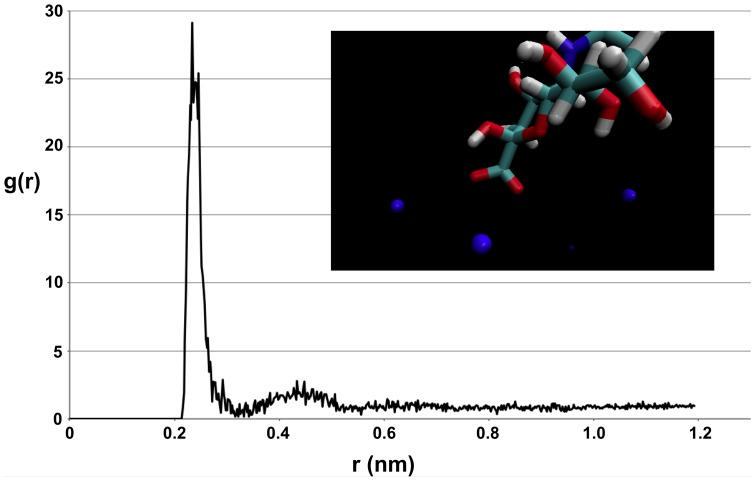
Oxygen-salt radial distribution function. Radial distribution function of the charged N-acetylneuraminic acid oxygen with the salt cations. The inset shows the salt ions (blue spheres) tending to cluster around the charged sugar site.

**Figure 4:**
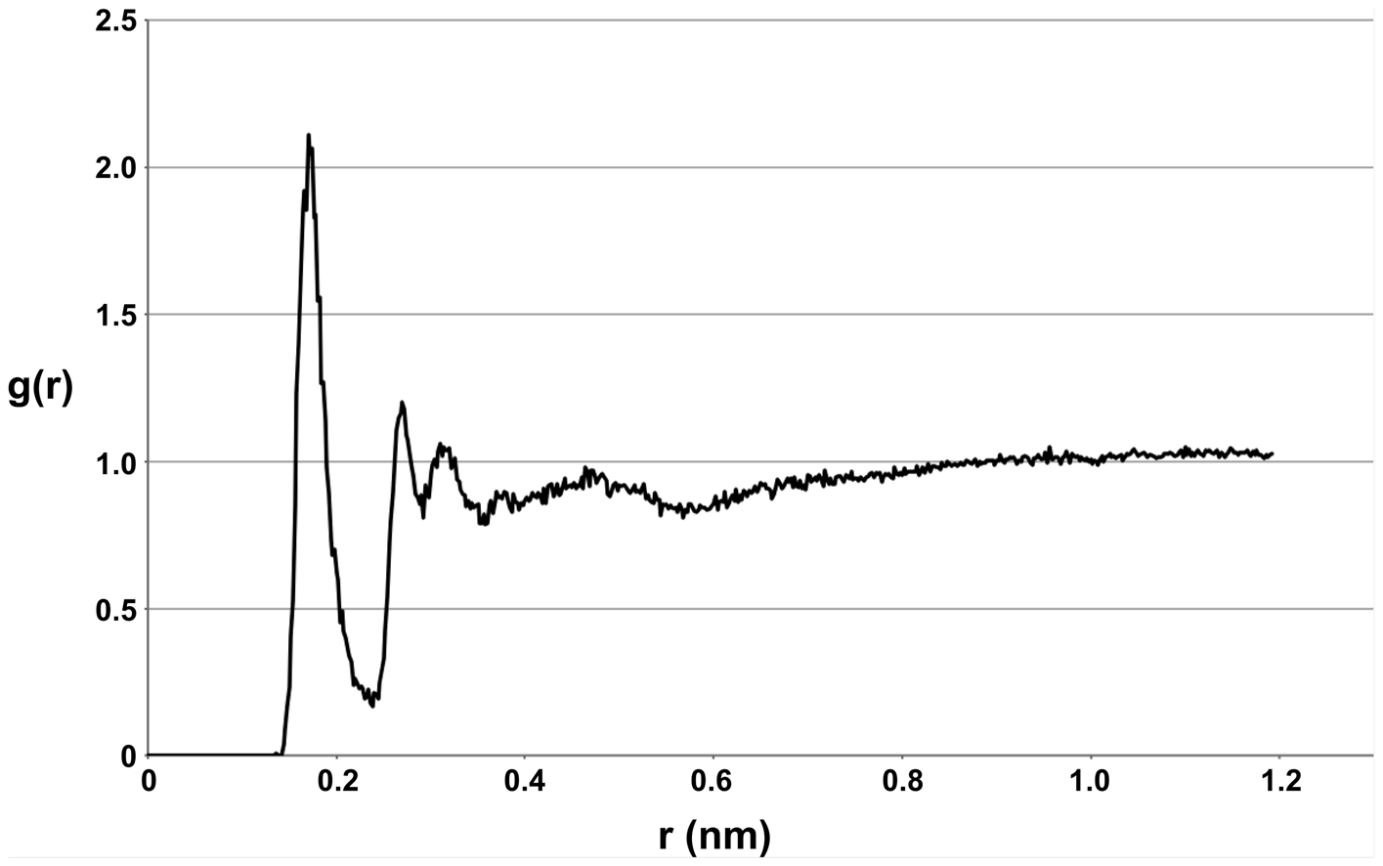
Oxygen-water radial distribution function. Radial distribution function of the charged N-acetylneuraminic acid oxygen with the water molecules.

To explain this decreased affinity for saline mucus, a radial distribution function (RDF) analysis was performed to determine the degree with which the salt ions interacts with the sugar molecule, N-acetylneuraminic acid. Figure 3 shows the RDF of the charged N-acetylneuraminic acid oxygen with the salt cations. The large peak at the small radial distance in this plot indicates that there is a high probability of finding the salt in the first coordination shell of the charged sugar site. The inset in Figure 3 also shows salt ions tending to cluster around the charged sugar site. Thus, it appears that the salt strongly favors and interacts with the sugar molecule. For comparison, a RDF of the charged N-acetylneuraminic acid oxygen with the water molecules was also computed (Figure 4), which shows characteristic coordination shells. However, the peak in the oxygen-water RDF is not nearly as large as that in the oxygen-salt RDF, indicating that the general sugar/water interactions are not nearly as strong as the sugar/salt interactions. Thus, it appears that the salt does indeed favor the sugar, and the addition of salt into the system has an appreciable effect on the chemical nature of the sugar molecule. In particular, it appears that the salt tends to increase the polarity and hydrophilic nature of the sugar. This essentially makes the saline mucus phase less preferable to the more hydrophobic amino acids, causing the reversal in odorant partitioning behavior observed in comparing Tables 2 and 3. Therefore, with the exception of glycine, the addition of salt into the system increases the affinity of the odorant molecules for the salt water phase, while also reducing their affinity for the saline mucus phase due to the interaction of the salt ions with N-acetylneuraminic acid. Compared with fresh water, this results in larger partition coefficients that tend to favor salt water over olfactory mucus.

## Discussion

The present study quantified water/mucus partition coefficients for four ecologically-relevant feeding stimulants in fish that have been shown to span a wide range of olfactory detection thresholds. In fresh water, all of the amino acid odorants were shown to possess a partition coefficient slightly less than one, meaning that each of the odorants prefer the mucus phase to water. In contrast, we found a reversal in odorant partitioning behavior in salt water, where, with the exception of glycine, each of the feeding stimulants prefer the salt water phase to olfactory mucus (i.e., a partition coefficient greater than one). This was due to the interaction of the salt ions with the amino acid odorant molecules in the water phase and N-acetylneuraminic acid in the simplified olfactory mucus phase. Thus, because the water/mucus odorant partition coefficients were found to differ between fresh water and salt water, we anticipate slightly varied odorant deposition patterns in the fish olfactory organ depending on the salinity of the underwater environment.

However, in either case (fresh water and salt water), we found that the variation of the calculated partition coefficients was approximately one order of magnitude. This is in stark contrast to air/mucus odorant partition coefficients, which can span up to six orders of magnitude [7], [18], [21], [24]. As a result, odorant deposition patterns in the nose of air-breathing animals are strongly dependent on the relative magnitude of the air/mucus partition coefficient, leading to odorant-dependent “imposed” patterning (see Introduction). However, given the comparatively weak variation of the water/mucus partition coefficients calculated in the present study, we anticipate relatively similar deposition patterns for most amino acid odorants in the fish olfactory chamber.

Such a hypothesis is consistent with the fact that ORNs are randomly distributed throughout the olfactory epithelium of fish [82]–[87]. That is, in contrast to air-breathing mammals that possess an “inherent” spatial distribution of ORNs in the olfactory epithelium that is utilized in concert with “imposed” odorant-dependent deposition patterns for olfactory discrimination (see Introduction), there is no spatial organization of ORNs in the fish olfactory epithelium. Our results, which indicate that there is unlikely to be significant odorant-dependent deposition patterning in the fish olfactory chamber, may partially explain why “the fish peripheral olfactory organ is somewhat different from other animals” [87] in its lack of ORN spatial organization. In other words, living in an aquatic environment may preclude appreciable imposed patterning in the fish olfactory chamber due to a limited range of water/mucus odorant partition coefficients, thereby eliminating the utility of spatial ORN segregation in the olfactory epithelium for coding odorant identity. Future odorant transport simulations in the olfactory chamber of the hammerhead shark are planned to investigate this hypothesis.
